# Diagnostic Value of Sirtuin-1 in Predicting Contrast-Induced Nephropathy After Percutaneous Coronary Intervention

**DOI:** 10.3390/jcm14113953

**Published:** 2025-06-03

**Authors:** Melis Ardic, Cuma Bulent Gul

**Affiliations:** 1 Department of Nephrology, Bursa Yuksek Ihtisas Training and Research Hospital, 16350 Bursa, Turkey; melisardicgh@gmail.com; 2 Departments of Nephrology, Faculty of Medicine, Bursa Uludag University, 16059 Bursa, Turkey

**Keywords:** Sirtuin-1, CI-AKI, post-PCI, renalism

## Abstract

**Objectives:** Contrast-induced acute kidney injury (CI-AKI) remains a frequent and serious complication after cardiac catheterization. Sirtuin-1 (SIRT1), a NAD+-dependent deacetylase, plays a central role in renal protection against ischemia-reperfusion injury, inflammation, and vascular dysfunction. We aimed to investigate whether serum SIRT1 levels could serve as an early diagnostic biomarker for CI-AKI. **Methods:** This prospective case-control study included 50 patients undergoing elective percutaneous coronary intervention (PCI) for stable angina. Serum SIRT1 levels were measured at baseline, 24 h, and 72 h post-PCI. The occurrence of CI-AKI was defined by a standard rise in serum creatinine, and patients were stratified accordingly. **Results:** Although SIRT1 levels tended to be lower in patients who developed CI-AKI (n = 17) compared to those without (n = 33), the differences were not statistically significant at any time point (*p* > 0.05). However, a significant between-group difference was observed in the 72-h change in SIRT1 levels (Δ0–72 h, *p* = 0.037), with a greater decline in the CI-AKI group. Multivariable logistic regression also revealed a trend-level inverse association between 72-h SIRT1 levels and CI-AKI (β = −0.536, *p* = 0.099). **Conclusions:** While SIRT1 is biologically plausible as a renal protective factor, our findings suggest that serial SIRT1 measurement may offer added value as a dynamic biomarker rather than a static diagnostic tool. Confirmatory trials incorporating serial SIRT1 measurements may help translate this molecular signal into clinically actionable tools for early detection of CI-AKI.

## 1. Introduction

Renalism, a term describing the hesitation to perform contrast-enhanced procedures due to the risk of acute kidney injury (AKI), is prevalent in cardiology practice, particularly in the context of interventional procedures. This apprehension can lead to delays in both diagnosis and treatment, potentially impacting patient outcomes negatively [1]. Even mild and transient forms of contrast-induced acute kidney injury (CI-AKI) have been associated with an increased risk of chronic kidney disease progression, cardiovascular complications, and mortality. As the use of percutaneous coronary interventions (PCI) continues to rise, the demand for early and reliable biomarkers of CI-AKI becomes increasingly critical. The pathophysiology of CI-AKI remains incompletely understood, but key mechanisms include renal vasoconstriction, impaired oxygen delivery, and hypoxia induced by contrast agents. These events trigger oxidative stress and the generation of reactive oxygen species (ROS), ultimately leading to tubular epithelial injury and nephrotoxicity [2].

Sirtuin 1 (SIRT1) is a NAD⁺-dependent deacetylase that regulates cellular responses to hypoxia, oxidative stress, and inflammation—key mechanisms involved in the pathogenesis of CI-AKI [3,4]. Through modulation of hypoxia-inducible factors (HIF-1α and HIF-2α) and endothelial nitric oxide synthase (eNOS), SIRT1 enhances vascular homeostasis and reduces renal tubular injury under stress conditions [4,5]. Preclinical studies have shown that SIRT1 activation protects against ischemia-reperfusion injury, suggesting its potential as a biomarker or therapeutic target in acute kidney injury [5,6]. However, its diagnostic utility in CI-AKI has not been adequately explored in clinical settings.

Given SIRT1’s established role in mitigating hypoxia-related renal injury and oxidative stress [4,5], we designed this study to evaluate whether serum SIRT1 levels could serve as an early biomarker for CI-AKI in patients undergoing PCI. We hypothesized that patients who develop CI-AKI would exhibit lower SIRT1 levels compared to those without renal injury, reflecting impaired cellular adaptation to contrast-induced hypoxia.

## 2. Method

### 2.1. Study Design

This was a prospective case-control study conducted at Bursa Yuksek Ihtisas Training and Research Hospital between July and November 2022. A total of 76 consecutive patients who underwent elective percutaneous coronary intervention (PCI) for stable angina were initially screened. After applying exclusion criteria and accounting for incomplete follow-up (n = 10), 50 patients were included in the final analysis. The study adhered to the STROBE guidelines for observational research.

### 2.2. Patient Selection

Adult patients aged 18 to 75 years who were scheduled for elective PCI due to stable angina and provided written informed consent were eligible for inclusion. Exclusion criteria were: (1) use of N-acetylcysteine prior to PCI (to ensure cohort homogeneity and avoid potential influence on SIRT1 levels), (2) presence of cardiogenic or septic shock, (3) documented acute or chronic kidney disease, (4) contrast exposure within the previous 48 h, and (5) inability to provide informed consent. Renal function was assessed using the CKD-EPI formula, and only patients with an estimated glomerular filtration rate (eGFR) ≥ 60 mL/min/1.73 m^2^ were included.

### 2.3. Study Procedure

Baseline demographic and clinical data—including age, sex, body mass index (BMI), and the presence of comorbid conditions such as diabetes mellitus, hypertension, and dyslipidemia—were recorded at enrollment. Diabetes was defined as a fasting plasma glucose level ≥ 126 mg/dL or the use of antidiabetic medication. Hypertension was defined as a systolic blood pressure > 140 mmHg and/or diastolic blood pressure > 90 mmHg, or current antihypertensive treatment for at least one year. Dyslipidemia was defined as a total serum cholesterol level > 220 mg/dL or the use of lipid-lowering agents.

### 2.4. Angiography Procedure

All procedures were performed via the standard femoral artery approach using conventional percutaneous coronary intervention (PCI) techniques. A nonionic, low-osmolar iodinated contrast agent—iopromide (Ultravist 370; Bayer, Istanbul, Turkey)—was administered at a volume of 50 to 80 mL per patient. Per institutional protocol, patients were advised to increase their oral fluid intake post-procedure to reduce the risk of CI-AKI.

### 2.5. Acute Kidney Injury Diagnosis

CI-AKI was defined according to widely used criteria as either an absolute increase in serum creatinine (sCr) of >0.5 mg/dL or a relative increase of ≥25% from baseline. Although these criteria are commonly used in CI-AKI research, they are distinct from the KDIGO definition of AKI, which includes an increase in sCr ≥ 0.3 mg/dL within 48 h or ≥1.5 times baseline within 7 days. Urine output criteria were not evaluated in this study. Serum creatinine levels were measured at baseline (prior to PCI), and at 24 and 72 h after contrast administration.

### 2.6. Serum SIRT1 Assay

Peripheral venous blood samples were obtained at baseline (prior to PCI), and at 24 and 72 h following contrast administration for the measurement of serum SIRT1 levels. Samples were immediately centrifuged at 4500 rpm for 10 min, and the serum was aliquoted and stored at −80 °C until analysis. SIRT1 concentrations were quantified using a commercially available human SIRT1 ELISA kit (SunRed Biotechnology, Shanghai, China) according to the manufacturer’s instructions. The intra-assay and inter-assay coefficients of variation were reported as 6% to 10%. The assay had a minimum detectable concentration of 0.306 ng/mL and a measurement range of 0.5–40 ng/mL.

### 2.7. Statistical Analysis

All statistical analyses were performed using SPSS version 21.0 (IBM Corp., Armonk, NY, USA). The distribution of continuous variables was assessed using the Kolmogorov–Smirnov test. Normally distributed variables were expressed as mean ± standard deviation (SD), while non-normally distributed variables were presented as median and interquartile range (IQR). Between-group comparisons were performed using the Student’s *t*-test or the Mann–Whitney U test, as appropriate. Categorical variables were analyzed using the Pearson χ^2^ test. A two-tailed *p*-value of <0.05 was considered statistically significant.

To assess the potential influence of age on serum SIRT1 levels, a linear regression analysis was conducted with age as the independent variable and SIRT1 levels at each time point (baseline, 24 h, and 72 h) as dependent variables.

The required sample size was calculated using G*Power version 3.1.9.7 [6]. Based on an alpha level of 0.05 and 80% statistical power, a minimum of 47 participants was deemed necessary to detect a significant difference in SIRT1 levels.

### 2.8. Ethics Statement

The study protocol was reviewed and approved by the Ethics Committee of Bursa Yuksek Ihtisas Training and Research Hospital (Approval number: 2011-KAEK-25 2022/01-06). All participants provided written informed consent prior to inclusion. The study was conducted in accordance with the principles of the Declaration of Helsinki.

## 3. Results

### 3.1. Baseline Characteristics of the Study Population

The demographic and clinical characteristics of the study cohort are presented in Table 1. A total of 50 patients were included in the final analysis, with 17 (34%) patients developing CI-AKI. The mean age was significantly higher in the CI-AKI group compared to the non-AKI group (69 ± 10 vs. 61 ± 13 years, *p* = 0.038). There were no statistically significant differences between the groups in terms of sex distribution, presence of diabetes or hypertension, or the use of renin–angiotensin system (RAS) blockers, beta-blockers, calcium channel blockers, statins, aspirin, or SGLT2 inhibitors (all *p* > 0.05).

### 3.2. Serum Creatinine and Incidence of CI-AKI

Serum creatinine (sCr) levels at baseline and follow-up time points are detailed in Table 2. At baseline, there was no significant difference in sCr between the CI-AKI and non-AKI groups (0.96 ± 0.21 mg/dL vs. 0.89 ± 0.18 mg/dL, respectively). However, sCr levels increased significantly in the CI-AKI group at both 24 h (1.11 ± 0.29 mg/dL, *p* = 0.015) and 72 h (1.55 ± 0.62 mg/dL, *p* < 0.001), confirming the diagnosis of CI-AKI based on predefined criteria.

### 3.3. Serum SIRT1 Levels at Baseline and Follow-Up

**Figure 1:**
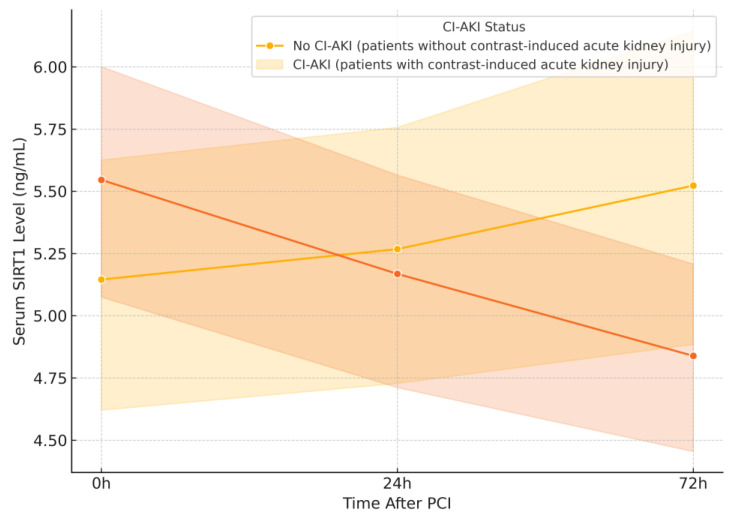
Changes in Serum SIRT1 Levels at 0, 24, and 72 h After PCI in Patients with and Without CI-AKI. The orange line represents the CI-AKI group (mean values), and the orange shaded area indicates the standard deviation. The yellow line represents the non-CI-AKI group (mean values). Serum SIRT1 levels were assessed at baseline, 24 h, and 72 h after PCI. Patients who developed CI-AKI showed a downward trend in SIRT1 levels, with a significant difference in the 72-h change compared to patients without CI-AKI (*p* = 0.037). Abbreviations: CI-AKI—contrast-induced acute kidney injury; PCI—percutaneous coronary intervention.

Serum SIRT1 levels measured at 0, 24, and 72 h after contrast administration are shown in Table 2 and Figure 1. While no statistically significant differences were observed between the CI-AKI and non-AKI groups at individual time points (*p* > 0.05), a downward trajectory was noted in the CI-AKI group (5.55 ± 2.08 at baseline, 5.17 ± 1.90 at 24 h, 4.84 ± 1.68 at 72 h). To further evaluate this pattern, we compared the temporal trends between groups. As illustrated in Figure 1, SIRT1 levels remained relatively stable in the non-AKI group, whereas a progressive decline was evident in those who developed CI-AKI. Although within-group comparisons were not significant (Friedman test, *p* > 0.05), the intergroup difference in 72-h change ΔSIRT1 (0–72 h) reached statistical significance (*p* = 0.037). Additionally, Pearson correlation analysis revealed no statistically significant association between age and SIRT1 levels at any time point (*p* > 0.60 for all), suggesting that age was not a contributing factor to SIRT1 variation in this cohort.

### 3.4. Groupwise Comparison of SIRT1 Levels Using Median Values

In addition to mean values, SIRT1 levels at each time point were analyzed using medians and interquartile ranges (IQR) to provide a more robust comparison between groups. As shown in Table S1, median SIRT1 levels at 0 h and 24 h were comparable between CI-AKI and non-AKI patients. However, at 72 h post-PCI, patients who developed CI-AKI had significantly lower SIRT1 levels (4.54 [3.53–5.74] vs. 4.97 [4.17–5.58], *p* = 0.037), further supporting a potential association between declining SIRT1 and renal injury. Detailed median and interquartile range values for each group are provided in Supplementary Table S1.

### 3.5. CRP and Other Biochemical Parameters

Table 3 presents additional biochemical parameters. While C-reactive protein (CRP) levels were higher in the CI-AKI group compared to the non-AKI group (18.0 ± 36.96 mg/dL vs. 7.26 ± 6.56 mg/dL), this difference was not statistically significant (*p* = 0.267). Lipid parameters, thyroid function (TSH), hemoglobin (Hgb), white blood cell (WBC) count, and troponin I levels also showed no significant differences between groups (all *p* > 0.05).

### 3.6. Multivariable Logistic Regression Analysis

To identify independent predictors of CI-AKI, a multivariable logistic regression model was constructed including age, comorbidities, SIRT1 levels at each time point, baseline creatinine, and commonly used cardiovascular medications. As presented in Table 4, most variables were not significantly associated with CI-AKI. However, SIRT1 levels at 72 h showed a negative association with CI-AKI development (β = −0.536, *p* = 0.099), suggesting a potential role for declining SIRT1 in identifying patients at risk. While this did not reach conventional significance, the direction and consistency of the effect support its biological plausibility.

## 4. Discussion

Despite the well-established protective role of Sirtuin 1 (SIRT1) in renal ischemia-reperfusion injury, inflammation, and oxidative stress, our study did not demonstrate a statistically significant association between serum SIRT1 levels and the development of CI-AKI following percutaneous coronary intervention (PCI). However, a more detailed temporal analysis revealed that SIRT1 levels declined significantly at 72 h in patients who developed CI-AKI, suggesting a delayed but potentially relevant biomarker response. While this result may initially appear negative, it nonetheless contributes meaningful insight to the emerging field of SIRT1-related nephroprotection. The observed trend toward lower SIRT1 levels in patients who developed CI-AKI supports the biological plausibility of SIRT1’s involvement in renal stress responses, even though statistical significance was not achieved in this sample. This highlights the importance of evaluating dynamic biomarker trajectories rather than relying solely on single-time-point measurements. This discrepancy between theoretical potential and clinical measurement underlines the complexity of translating molecular pathways into usable biomarkers, particularly in acute and dynamic clinical settings such as contrast-induced nephropathy.

Preclinical studies have consistently shown that SIRT1 activation confers renal protection by attenuating tubular epithelial cell apoptosis, enhancing mitochondrial function, and suppressing pro-inflammatory pathways [4,5,7]. In murine models of ischemia-reperfusion and cisplatin-induced nephrotoxicity, both pharmacologic activators of SIRT1 (e.g., resveratrol, SRT1720) and genetic overexpression led to improved renal outcomes [3,4]. Specifically, in CI-AKI models induced by iodinated contrast agents, SIRT1 upregulation has been associated with reduced oxidative stress and preserved tubular architecture [3]. These findings provided a strong rationale for our hypothesis that SIRT1 could serve as an early biomarker of CI-AKI. Our observation of a significant decline in serum SIRT1 levels at 72 h in CI-AKI patients aligns with these preclinical models, where impaired SIRT1 activity has been linked to greater renal vulnerability. However, despite the biological plausibility and temporal sampling post-contrast exposure, our study did not find significant differences in serum SIRT1 levels between CI-AKI and non-CI-AKI groups. This may reflect the complex interplay between systemic and localized SIRT1 expression, or the dynamic and possibly transient nature of SIRT1 modulation in acute injury settings. This observed variability may also reflect inter-individual differences in metabolic state, subclinical inflammation, and renal response to hypoxia, all of which can influence circulating SIRT1 levels.

In addition, ischemia-reperfusion (I/R) injury—a key contributor to CI-AKI pathogenesis—is known to trigger tubular epithelial cell (TEC) apoptosis. SIRT1 has been shown to attenuate I/R-induced renal damage by reducing oxidative stress and inhibiting apoptosis. Gong et al. demonstrated that SIRT1 activation alleviates TEC apoptosis and oxidative injury under hypoxic conditions [7], while He et al. reported that SIRT1 deficiency sensitizes renal medullary cells to oxidative stress [4]. In our study, the observed reduction in SIRT1 levels at 72 h in the CI-AKI group may reflect an insufficient endogenous response to contrast-induced hypoxic stress, consistent with findings from I/R animal models. These findings support the notion that lower circulating SIRT1 levels in our CI-AKI group may reflect impaired endogenous defense against I/R injury.

SIRT1 has also been implicated in age-related physiological decline, with prior studies suggesting a gradual reduction in SIRT1 expression with advancing age [8]. For instance, Borsky et al. demonstrated lower SIRT1 levels in older adults, correlating with markers of biological aging. In our study, the mean age of patients in the CI-AKI group was significantly higher than in the non-AKI group, raising the possibility that age-related SIRT1 depletion could have influenced our results. However, in our multivariable regression analysis, age was not independently associated with CI-AKI, nor did it significantly affect SIRT1 levels at any time point. This lack of association may be explained by the relatively narrow age distribution within our sample, the presence of shared atherosclerotic risk factors that may have blurred biological age differences, or the limited power to detect subtle effects. It is also possible that SIRT1 activity, rather than circulating levels, plays a more critical role in mediating age-dependent susceptibility to kidney injury, a distinction not captured by ELISA-based quantification.

Inflammation is a well-recognized contributor to the development of CI-AKI, and SIRT1 has been shown to exert anti-inflammatory effects through multiple mechanisms, including inhibition of the NF-κB signaling pathway and deacetylation of high-mobility group box 1 (HMGB1) protein [9,10]. In experimental models, these pathways have been implicated in modulating cytokine production, leukocyte infiltration, and tubular injury. In our study, CRP levels—a surrogate marker of systemic inflammation—were higher in the CI-AKI group, although the difference did not reach statistical significance. This non-significant elevation may still reflect a heightened inflammatory response in susceptible individuals. Importantly, SIRT1 levels were not inversely correlated with CRP, which may indicate that circulating SIRT1 concentrations do not adequately reflect tissue-level anti-inflammatory activity or the temporal dynamics of acute inflammation. It is also plausible that the timing of sample collection did not align with the peak inflammatory response or that SIRT1’s anti-inflammatory role is more pronounced at the tissue level rather than in the circulation.

This study has several limitations that should be considered when interpreting the results. First, while the sample size was adequate for detecting changes in SIRT1 levels, it may have been underpowered to assess secondary associations such as the impact of age or inflammation. Second, SIRT1 levels were measured in peripheral serum, which may not reflect local renal tissue activity, especially given the intracellular nature of its regulatory functions. Moreover, the statistical significance observed only at the 72-h time point may indicate that SIRT1’s prognostic value is time-dependent and requires sequential measurement to be clinically meaningful. Third, as a single-center study, generalizability to other populations may be limited. Finally, the absence of dynamic markers, such as urinary biomarkers or serum markers (e.g., NGAL, IL-18), along with the lack of direct tissue-level assessment, limits the mechanistic interpretation of our findings. Despite these limitations, the identification of a temporal decline in SIRT1 levels among CI-AKI patients provides a valuable signal and supports the need for further research evaluating SIRT1 as a component of dynamic biomarker panels for acute kidney injury.

## 5. Conclusions

Although serum SIRT1 levels at isolated time points were not significantly associated with the development of CI-AKI, our analysis revealed a significant decline in SIRT1 levels at 72 h in affected patients. This temporal pattern suggests that SIRT1 may hold potential as part of a dynamic biomarker panel rather than as a static, standalone indicator. Given SIRT1’s established role in renal protection, inflammation, and oxidative stress, further prospective studies incorporating serial measurements and functional endpoints are warranted to better define its diagnostic and prognostic utility in acute kidney injury.

## Figures and Tables

**Table 1:** General characteristics of patients.

	Without AKI (n = 33)	with CI-AKI (n = 17)	*p*
Age(years)	61 ± 13	69± 10	0.038
Female gender [n (%)]	10 (30)	7 (41)	0.534
Diabetes [n (%)]	13 (40)	7 (41)	0.904
Hypertension [n (%)]	18 (55)	14 (82)	0.550
RAS Blocker [n (%)]	10 (30.3)	8 (47.1)	0.242
Beta-blocker [n (%)]	13 (39.4)	7 (41.2)	0.903
Ca-Channel Blocker [n (%)]	5 (15.2)	6 (35.3)	0.151
Statins [n (%)]	4 (12.1)	4 (23.5)	0.419
Aspirin [n (%)]	11 (33.3)	7 (41.2)	0.585
Clopidogrel [n (%)]	6 (18.2)	1 (5.9)	0.398
SGLT2-I [n (%)]	10 (30.3)	8 (47.1)	0.264

*p*-values were calculated with the χ^2^ test or the Student’s *t*-test, as appropriate.

**Table 2:** Serum SIRT-1 and creatinine values in patients with and without CI-AKI.

	Without AKI (n = 33)		with CI-AKI (n = 17)	
	Mean ± SD		Mean ± SD	
SIRT-1 (0 h)	5.15	±2.87	5.55	±2.08
SIRT-1 (24 h)	5.27	±3.21	5.17	±1.90
SIRT-1 (72 h)	5.52	±3.75	4.84	±1.68
sCr (0 h)	0.89	±0.18	0.96	±0.21
sCr (24 h)	0.89	±0.21	1.11	±0.29
sCr (72 h)	0.91	±0.18	1.55	±0.62

SIRT-1; sirtuin-1, sCr; serum creatinine, SD; standard deviation. In the CI-AKI group, mean serum creatinine increased from 0.96 ± 0.21 mg/dL at baseline to 1.11 ± 0.29 mg/dL at 24 h and 1.55 ± 0.62 mg/dL at 72 h. Compared to baseline, these changes were statistically significant (*p* = 0.015 and *p* = 0.0001, respectively).

**Table 3:** Biochemical values of the patients.

	Without AKI (n = 33) Mean ± SD	with CI-AKI (n = 17) Mean ± SD	*p*
CRP (mg/dL)	7.26 ± 6.56	18 ± 36.96	0.267
Cholesterol (mg/dL)	196 ± 42	199 ± 55	0.845
HDL (mg/dL)	41 ± 10	45 ± 16	0.363
LDL (mg/dL)	125 ± 30	132 ± 50	0.647
Triglyceride (mg/dL)	160 ± 116	127 ± 90	0.285
TSH (uIU/mL)	1.10 ± 0.87	1.95 ± 2.48	0.249
Hgb (g/dL)	13.21± 1.86	13.15 ± 2.14	0.927
WBC (10^3^/mL)	11.12 ± 2.95	11.39 ± 5.50	0.824
Troponin I (ng/L)	22.42 ± 72.66	4.17 ± 8.65	0.319

CRP; C reactive protein, HDL; high-density lipoprotein, LDL; low-density lipoprotein, TSH; thyroid stimulating hormone, Hgb; hemoglobin, WBC; white blood cells. *p* values were calculated with the Mann-Whitney U or the Student’s *t*-test, as appropriate.

**Table 4:** Multivariable Logistic Regression for Predictors of CI-AKI.

Variable	β Coefficient	Standard Error	*p*-Value	95% CI (Lower–Upper)
Age	+0.040	0.034	0.238	−0.026 to +0.105
Diabetes	−0.164	0.868	0.850	−1.866 to +1.538
Hypertension	+1.390	1.008	0.168	−0.587 to +3.366
SIRT1 at 0 h	+0.378	0.322	0.242	−0.254 to +1.010
SIRT1 at 24 h	+0.216	0.338	0.522	−0.446 to +0.878
SIRT1 at 72 h	−0.536	0.325	0.099	−1.172 to +0.100
Baseline Cr	+1.547	2.107	0.463	−2.583 to +5.677
ACEi/ARB	−0.067	0.915	0.942	−1.859 to +1.726
Beta-blocker	−0.847	1.011	0.402	−2.827 to +1.134
Loop diuretic	+1.181	2.034	0.562	−2.806 to +5.167
Thiazide	−0.061	1.238	0.961	−2.487 to +2.365
SGLT2 inhibitor	+1.894	1.738	0.276	−1.512 to +5.300
Statin	+0.555	1.180	0.638	−1.757 to +2.868

Logistic regression results showing the association between baseline clinical characteristics, serum SIRT1 levels at 0, 24, and 72 h, and the development of CI-AKI. Regression coefficients (β), standard errors, *p*-values, and 95% confidence intervals (CI) are presented. While most variables were not significantly associated with CI-AKI, SIRT1 levels at 72 h exhibited a trend-level inverse association (*p* = 0.099). Abbreviations: CI-AKI—contrast-induced acute kidney injury; CI—confidence interval; SIRT1—sirtuin 1.

## Data Availability

The original contributions presented in this study are included in the article/Supplementary Material. Further inquiries can be directed to the corresponding author.

## Supplementary Materials

The following supporting information can be downloaded at: https://www.mdpi.com/article/10.3390/jcm14113953/s1, Table S1. Median Serum SIRT1 Levels at 0, 24, and 72 h After PCI, Stratified by CI-AKI Status.
