# Supplementary material for: Diagnostic Value of Sirtuin-1 in Predicting Contrast-Induced Nephropathy After Percutaneous Coronary Intervention
Source: J Clin Med. 2025 Jun 3;14(11):3953. doi: 10.3390/jcm14113953 (PMC12156447; doi:10.3390/jcm14113953)
Supplement: Supplementary file 1 [file jcm-14-03953-s001.zip › jcm-3669220-supplementary.pdf]

Supplementary Table S1. Median Serum SIRT1 Levels at 0, 24, and 72 Hours After PCI, Stratified by CI-AKI Status.

| ***Group*** | ***SIRT1 at 0h*** | ***SIRT1 at 24h*** | ***SIRT1 at 72h*** |
|---|---|---|---|
| *No CI-AKI* | 4.57 [3.79–5.29] | 4.88 [3.50–5.86] | 4.97 [4.17–5.58] |
| *CI-AKI* | 4.69 [4.44–6.45] | 4.88 [3.89–5.50] | 4.54 [3.53–5.74] |
| *p-value* | – | 0.395 | 0.037 |

SIRT1 values are presented as median [interquartile range]. Between-group comparisons were performed using the Mann–Whitney U test. Although no significant differences were observed at baseline or 24 hours, a statistically significant difference was noted at 72 hours, with lower SIRT1 levels in the CI-AKI group ($p = 0.037$). **Abbreviations:** CI-AKI – contrast-induced acute kidney injury; PCI – percutaneous coronary intervention.
